# Uncertainty-Guided Active Learning for Access Route Segmentation and Planning in Transcatheter Aortic Valve Implantation

**DOI:** 10.3390/jimaging11090318

**Published:** 2025-09-17

**Authors:** Mahdi Islam, Musarrat Tabassum, Agnes Mayr, Christian Kremser, Markus Haltmeier, Enrique Almar-Munoz

**Affiliations:** 1 Department of Radiology, Medical University of Innsbruck, 6020 Innsbruck, Austria; mahdiislam@iut-dhaka.edu (M.I.); tabassum67@iut-dhaka.edu (M.T.); a.mayr@i-med.ac.at (A.M.); christian.kremser@i-med.ac.at (C.K.); 2 Department of Mathematics, University of Innsbruck, 6020 Innsbruck, Austria; markus.haltmeier@uibk.ac.at

**Keywords:** active learning, vessel diameter quantification, aortic segmentation, TAVI planning, cardiovascular magnetic resonance

## Abstract

Transcatheter aortic valve implantation (TAVI) is a minimally invasive procedure for treating severe aortic stenosis, where optimal vascular access route selection is critical to reduce complications. It requires careful selection of the iliac artery with the most favourable anatomy, specifically, one with the largest diameters and no segments narrower than 5 mm. This process is time-consuming when carried out manually. We present an active learning-based segmentation framework for contrast-enhanced Cardiac Magnetic Resonance (CMR) data, guided by probabilistic uncertainty and pseudo-labelling, enabling efficient segmentation with minimal manual annotation. The segmentations are then fed into an automated pipeline for diameter quantification, achieving a Dice score of 0.912 and a mean absolute percentage error (MAPE) of 4.92%. An ablation study using pre- and post-contrast CMR showed superior performance with post-contrast data only. Overall, the pipeline provides accurate segmentation and detailed diameter profiles of the aorto-iliac route, helping the assessment of the access route.

## 1. Introduction

Aortic stenosis (AS), a progressive narrowing of the aortic valve opening, represents the most prevalent valvular heart disease in the Western world, affecting approximately 12.4% of individuals over 75 years old, with severe AS present in about 3.4% of this population [1]. This condition, often caused by age-related calcification and scarring, restricts blood flow from the heart, yet many elderly patients are considered too high-risk for traditional open-heart surgery. For these patients, transcatheter aortic valve implantation (TAVI), a minimally invasive procedure, has emerged as the gold-standard treatment [2]. Successful TAVI planning relies on precise pre-procedural imaging to assess the size of the valve prosthesis and the aortoiliofemoral access route. The second involves evaluating the diameter of the iliac arteries to determine their suitability for catheter insertion. Specifically, arteries with diameters below **5 to 5.5 mm** are associated with a heightened risk of vascular complications, including iliac artery rupture, which can be fatal [3]. The gold-imaging modality for this task is contrast-enhanced Computed Tomography Angiography (CTA) [4].

However, the reliance on CTA presents a significant clinical challenge. A substantial portion of the TAVI patient population suffers from pre-existing renal impairment, with reported prevalence rates of chronic kidney disease (CKD) as high as 70% [5]. The iodinated contrast agents required for CTA can be nephrotoxic and contraindicated in these patients, creating a critical need for iodinated-free imaging alternatives. Cardiovascular Magnetic Resonance (CMR) has emerged as a promising solution, capable of providing the necessary anatomical and functional details for TAVI planning [6]. Studies comparing non-contrast MRA with CTA have found strong correlations in vessel diameter measurements, resulting in comparable decisions regarding transfemoral access capacity [7].

Post-contrast CMRs enhance the visibility of vascular structures, making it easier to extract meaningful anatomical insights from segmentation. Consequently, CMR-based TAVI planning holds strong potential for providing accurate measurements of critical anatomical structures like aorta and iliacs, while avoiding the risks associated with contrast exposure.

One of such insights can be the diameter of aortic structures, especially the iliac arteries, which are crucial for selecting the optimal access route during TAVI. These diameters can be derived from segmentation masks of the specific structures. However, most previous studies on aortic structure measurement have primarily focused on the aorta alone [8]. Although some recent pipelines, such as ARVA, have extended their measurements to include iliac arteries, they remain limited to maximum diameters or volumetric evaluation at discrete segments rather than continuous profiling along the entire aorto-iliac path [9]. Moreover, existing works often calculate diameters only at specific anatomical landmarks (e.g., the aortic root or ascending aorta), rather than providing a continuous assessment along the full vessel trajectory [10,11]. To the best of the authors’ knowledge, while some studies such as ARVA have included iliac diameter measurements, no work has specifically addressed the continuous computation of iliac artery diameters along the access route to support transfemoral TAVI planning. Given that CTA remains the most common modality for TAVI procedures, it has been widely used for training deep learning-based aortic segmentation algorithms. Several studies have extended this work to automatic measurement of aortic annulus diameters and morphological biomarkers of the aortic root for pre-procedural planning [12,13]. Building on these diameter-focused works, recent research has shifted toward developing end-to-end automated CT pipelines that not only segment the aorta but also extract multiple anatomical measurements required for TAVI planning.

Recent literature highlights progress in automated pipelines for TAVI planning. Methods such as TAVI-PREP and 4TAVR have shown that deep learning can reliably extract multiple anatomical measurements and perform full CT-based planning with accuracy comparable to clinical experts [14,15].

Central to any planning pipeline is the accurate segmentation of the aorta and iliac arteries. Deep learning models like DeepLabv3+ have proven effective for this task in both CT and CMR contexts. One study successfully applied such models for real-time aortic valve detection from cardiac CT, indicating the feasibility of automated segmentation [16]. Recent architectures like CIS-UNet further improve multi-class segmentation of the aorta and its branches by integrating convolutional and transformer-based modules, enabling accurate delineation of complex vascular structures in CTA volumes [17]. However, a persistent challenge remains in segmenting smaller, more intricate structures like the iliac arteries, particularly from CMR data where image quality or contrast maybe limited compared to CTA.

There have been few works using CMR for aortic structure measurement in TAVI patients, and to the best of the authors’ knowledge, there are no publicly available CMR datasets specifically curated for segmentation of aortic structures and diameter estimation for transfemoral access route planning. This limitation is partly due to the “annotation bottleneck,” since supervised deep learning models require large, high-quality labelled datasets, which are scarce in CMR. To address this, semi-supervised and active learning (AL) strategies have been explored in related contexts. For example, quality-aware SSL approaches [18] and recent foundation models like MedSAM2 [19] aim to reduce the reliance on manual labels, while predictive-accuracy-based active learning frameworks [20] and hybrid methods such as test-time augmentation for active learning [21] or selective uncertainty-based active learning [22] combine pseudo-labelling with uncertainty-aware sampling to maximize efficiency. Although most of these methods have been applied to CT or other segmentation tasks (e.g., aortic aneurysm), they provide promising directions for CMR-based TAVI planning.

To address the challenges of manual iliac artery assessment in TAVI planning, this paper proposes a comprehensive framework for the semi-automated analysis of both pre- and post-contrast CMR images, with a focus on annotation efficiency. The contributions of this work are fourfold:

- An active learning pipeline for aortic structure segmentation is introduced, designed to achieve high accuracy while significantly reducing the manual annotation effort compared with traditional fully supervised methods. Unlike prior segmentation works that focused on CTA or abdominal aorta only, this represents one of the first applications for CMR-based TAVI planning.
- A robust uncertainty estimation strategy is incorporated to guide sample selection, whereby the most informative cases are queried for expert review and high-confidence predictions are used for pseudo-labelling. This hybrid strategy optimizes the use of limited annotation budgets, addressing the annotation bottleneck that has not been previously explored in the TAVI context.
- A complete workflow is developed that extends beyond segmentation to include automated centerline extraction and a hybrid diameter calculation strategy. In contrast to existing studies that report only landmark-based or discrete diameter measurements, this study enables continuous profiling of vessel diameters along the aorto-iliac path, which has not yet been addressed in prior literature.
- Using a dataset of paired pre- and post-contrast scans, a modality ablation study is performed to systematically evaluate the distinct and combined contributions of each imaging modality. To the best of authors’ knowledge, this is the first such evaluation in the context of CMR-based TAVI planning.

The rest of this paper is organized as follows. Section 2 describes the dataset. Section 3 presents the proposed method, with details provided within the section. Section 4 reports the experimental results, covering the diameter profiles, the active learning performance, and the ablation results. Section 5 explains the important findings of the results.

## 2. Dataset

The dataset used in this in-house retrospective study includes 337 CMR scans in DICOM format: 166 native (pre-contrast) and 171 post-contrast acquired during routine pre-TAVI assessments. For this study, we specifically used the post-contrast CMR scans acquired within the framework of the TAVR-CMR trial [23], a prospective, randomized, open-label, noninferiority trial conducted at two Austrian heart centers (Medical University of Innsbruck and University Teaching Hospital Wels-Grieskirchen) between 11 September 2017 and 16 December 2022. Imaging in this trial was performed on a 1.5 T clinical scanner (Magnetom AVANTOfit, Siemens, Munich, Germany) using a standardized protocol optimized for visualizing the aortic root and iliofemoral access vessels. The imaging protocol included a non-contrast-enhanced, ECG-triggered, navigator-gated, free-breathing 3D whole-heart steady-state free precession (SSFP) sequence covering the left ventricular outflow tract to the ascending aorta, as well as coronal 3D FLASH magnetic resonance angiography (MRA) performed before and after contrast administration. Contrast-enhanced scans were acquired following intravenous injection of gadobutrol (0.2 mmol/kg, Gadovist, Schering) at a rate of 2 mL/s, achieving coverage from the supra-aortic branches down to the femoral arteries. This acquisition strategy enabled high-quality visualization of both the central aortic root and peripheral access routes, facilitating accurate segmentation of the aorta and iliac arteries for TAVI planning.

**Figure 1:**
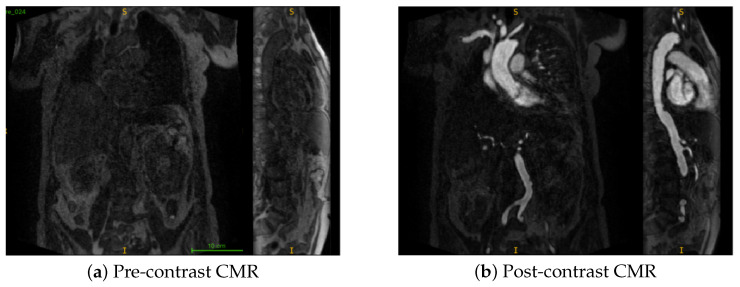
Coronal and sagittal views of representative samples from the dataset. Here, S denotes the superior (upper) direction and I denotes the inferior (lower) direction in the image. (**a**) Pre-contrast CMR (**b**) Post-contrast CMR.

The original CMR scans had dimensions of 330 × 112 × 352 (x–y–z), with a voxel resolution of approximately 1.28 × 1.25 × 1.28 mm^3^ and an LPI (Left–Posterior–Inferior) orientation; all scans were subsequently reoriented to RAS (Right-Anterior-Superior) and resampled to isotropic 1 × 1 × 1 mm^3^ during preprocessing. Examples are shown in Figure 1. All scans were converted from DICOM to NIfTI format, reoriented to the RAS (Right–Anterior–Superior) coordinate system, and resampled to 1 × 1 × 1 mm^3^ voxel spacing using trilinear interpolation.

## 3. Methods

This section details the methods employed in this research, encompassing Section 3.1–Section 3.3. Furthermore, it describes the quantitative criteria used.

### 3.1. Vessel Diameter Quantification

**Figure 2:**
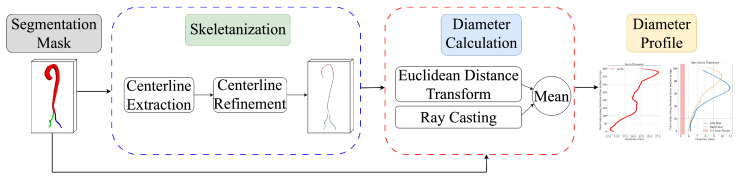
Overview of the automated vessel diameter quantification pipeline.

The pipeline (Figure 2) shows how aortic diameters are measured from 3D segmentation masks through centerline extraction and diameter measurement.

#### 3.1.1. Centerline Extraction

From the vessel segmentation mask $M_{RAS}$ (converted to $M_{ZYX}$), the centerline is extracted:

- **Morphological Skeletonization**: The 3D binary mask $M_{ZYX}$ is iteratively thinned by successively removing boundary voxels while preserving the overall topology, resulting in a 1-voxel-wide skeleton $S_{ZYX}$ that represents the medial axis of the structure.
- **Largest Connected Component (LCC)**: Only the LCC of $S_{ZYX}$, $S_{LCC}$, is retained to remove noise/irrelevant branches.
- **Graph Representation and Path Finding**: $S_{LCC}$ is converted to a graph G=(V,E) using ‘skan.skeleton_to_csgraph’. Endpoints $V_{EP}\subset V$ are identified. The principal centerline is defined as the longest shortest path between any two endpoints in *G*, obtained with Dijkstra’s algorithm [24].
- **Centerline Ordering**: The sequence of ZYX coordinates $C={\left\{\left(z_{k},y_{k},x_{k}\right)\right\}}_{k=1}^{N_{p}}$ represents the $N_{p}$ points along the extracted longest path of the skeleton. To enforce a consistent orientation, the path is ordered such that the Z-coordinate of the first point ($z_{1}$) is less than or equal to the Z-coordinate of the last point ($z_{N_{p}}$), i.e., $z_{1}\leq z_{N_{p}}$. This ensures a robust and reproducible inferior-to-superior progression (or an equivalent direction depending on the Z-axis definition) when traversing the centerline.

#### 3.1.2. Centerline Refinement

1. **Smoothing**: To mitigate voxel discretization artifacts, Y and X coordinates of the raw centerline *C* are smoothed using a 1D uniform filter as Equation (1).
  (1) $$y_{k}^{\prime }=\mathrm{uniform}\_\mathrm{filter}1d\left(y_{k},\mathrm{win}\right),\;x_{k}^{\prime }=\mathrm{uniform}\_\mathrm{filter}1d\left(x_{k},\mathrm{win}\right)$$
  Sudden coordinate jumps exceeding a threshold are corrected by local averaging. The smoothed centerline is $C^{\prime }={\left\{\left(z_{k},y_{k}^{\prime },x_{k}^{\prime }\right)\right\}}_{k=1}^{N_{p}}$.
2. **Endpoint Correction**: For an initial fraction of $C^{\prime }$ points, 5%, their Y and X coordinates are adjusted to the Center of Mass (CoM) of the vessel mask $M_{ZYX}$ in the corresponding Z-slice $z_{k}$. This ensures the centerline robustly starts within the vessel lumen at its ends, yielding $C^{\prime \prime }$.

#### 3.1.3. Diameter Calculation

For each point $P_{k}=\left(z_{k},y_{k},x_{k}\right)$ on the refined centerline $C^{\prime \prime }$, the local vessel diameter is estimated using a hybrid approach, with methodological grounding in Distance Transform methods [25,26] and ray-casting strategies for vascular quantification [27,28,29].

1. **Distance Transform (DT) Diameter**: A 3D Euclidean Distance Transform (EDT) is computed on $M_{ZYX}$ considering voxel spacing *S*. $D_{DT,k}=2\;\cdot \;EDT\left(P_{k}\right)$ gives the initial diameter estimate.
2. **Ray-Casting Diameter**:
  - **Local Tangent:** A local tangent vector ${\vec{T}}_{k}$ at $P_{k}$ is computed using finite differences between neighboring centerline points.
  - **Orthogonal Plane Definition:** A plane orthogonal to ${\vec{T}}_{k}$ is defined.
    – If ${\vec{T}}_{k}$ is nearly parallel to the Z-axis (i.e., $|{\vec{T}}_{k}\;\cdot \;\hat{z}|>\cos\theta_{thresh}$), the YX-plane at $z_{k}$ is used.
    – Otherwise, two orthogonal vectors ${\vec{U}}_{k}$ and ${\vec{V}}_{k}$ are computed that span the plane perpendicular to ${\vec{T}}_{k}$ (e.g., using the Frisvad method), ensuring numerical stability and orthogonality.
  - **Ray Casting:** A set of $N_{rays}$ (e.g., 8) rays are cast from $P_{k}$ within this plane. Each direction of ray ${\vec{d}}_{ki}$ is defined as a linear combination of ${\vec{U}}_{k}$ and ${\vec{V}}_{k}$, or lies within the YX plane if the Z-parallel condition holds.
  - **Boundary Detection:** For each ray originating at $P_{k}$ and extending in directions $\pm {\vec{d}}_{ki}$, the exit points where the ray leaves the vessel mask $M_{ZYX}$ are detected, denoted as $B_{ki}^{+}$ and $B_{ki}^{-}$.
  - **Ray Diameter:** The diameter along each ray is computed as Equation (2):
    (2) $$D_{ray,ki}=∥\left(B_{ki}^{+}-B_{ki}^{-}\right)⊙S{∥}_{2},$$
    where ⊙ denotes element-wise multiplication with voxel spacing *S*.
3. **Combined Diameter**: The “raw” diameter is defined as Equation (3)
  (3) $$D_{raw,k}=\frac{D_{DT,k}+\mathrm{median}\left(\left\{D_{ray,ki}\right\}\right)}{2}.$$
  If missing values in $\left\{D_{raw,k}\right\}$ are encountered, they are linearly interpolated. Finally, a Savitzky–Golay filter (polynomial order 3, window length up to 51 points) is applied to obtain the final smoothed diameter profile $\left\{D_{final,k}\right\}$.

### 3.2. Active Learning Implementation

**Figure 3:**
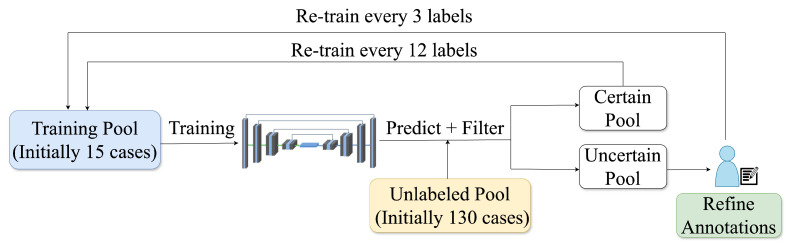
The active learning cycle includes training, inference on unlabelled data, uncertainty-based ranking, and sample selection, with uncertain cases manually annotated and certain ones pseudo-labelled to expand the training set.

This study used active learning (AL) to develop a robust segmentation pipeline of the aortic and iliac arteries with minimal manual annotation. AL uses iterative training by selectively including challenging, uncertain samples to accelerate performance and achieve high accuracy with fewer labels [30]. For AL, 171 post-contrast CMR scans were used due to their enhanced visibility of the vessel. These were randomly shuffled and divided into 145 training and 26 holdout test samples. The training set was further divided into an initial training pool (15 randomly selected samples annotated by an expert) and an unlabelled pool for subsequent AL iterations. An overview of the process can be seen in Figure 3.

For the segmentation backbone, nnUNet V2 with the Residual Encoder U-Net (ResUNet) preset [31] was used. Compared to the standard 3D U-Net, the ResUNet introduces residual connections within the encoder blocks, which substantially improves gradient flow, stabilizes deeper architectures, and has been shown to improve segmentation performance across diverse datasets. The ResUNet architecture was automatically adapted to the properties of the dataset (target spacing, patch size, anisotropy) by the nnUNet planning module, including the number of layers, the size of the convolutional kernel, the structure of groups and the configurations of the feature map. Ground truth masks of the aorta and iliac arteries, manually annotated by an expert, were used for supervision. The training configuration employed in each iteration is summarized in Table 1.

#### Uncertainty Estimation

To guide sample selection in active learning, a robust uncertainty estimation strategy was implemented using the probabilistic output of the U-Net. For each unlabelled case, probability maps ($p_{j}$) were generated for Aorta, Left Iliac, and Right Iliac arteries. Uncertainty was calculated per class using these probabilities for voxels with $p_{j}>0.01$. A composite Case Uncertainty Score ($S_{\mathrm{case}}$) was formulated for iliac arteries:

(4) $$S_{c}={\bar{H}}_{c}+\left(1-\frac{F_{\mathrm{very}\_\mathrm{confident},c}}{100}\right)$$

Here, ${\bar{H}}_{c}$ is the average binary entropy, and $F_{\mathrm{very}\_\mathrm{confident},c}$ denotes the fraction of very confident voxels, defined as the percentage of voxels with predicted probabilities greater than 0.9. The final $S_{\mathrm{case}}$ was the average across relevant iliac artery classes: $S_{\mathrm{case}}=\mathrm{mean}\left(S_{\mathrm{LI}},S_{\mathrm{RI}}\right)$. Higher $S_{\mathrm{case}}$ indicated greater uncertainty, guiding the selection of the 3 most uncertain (underwent manual annotation) and 12 most certain samples (pseudo-labels) for subsequent AL iterations.

This iterative cycle of training, inference, uncertainty-based selection, and selective re-annotation was repeated until the test performance plateaued.

### 3.3. Multimodal Ablation Study

After the uncertainty-based AL process plateaued, we retained all manually masks along with the pseudo-labelled ones to perform ablation studies and identify the best-performing configuration. Two segmentation models, 3D U-Net and UMamba [32] were each trained for 100 epochs across four distinct configurations. The first configuration used only the post-contrast CMR modality as input. The second configuration combined both pre- and post-contrast co-registered images to provide complementary information. The third and fourth configurations repeated the first two, respectively, but with the addition of inferred masks from the unseen pool of the active learning process. These inferred masks were generated using the final AL model after its performance had plateaued, and were included as pseudo-labelled training data to further enhance the models’ learning.

Evaluation was performed on the held-out test set, with performance reported separately for each class, model and input data configuration. The segmentation performance was assessed using the Dice Similarity Coefficient (DSC) and Mean Absolute Percentage Error (MAPE).

## 4. Results

### 4.1. Vessel Diameter Profile

**Figure 4:**
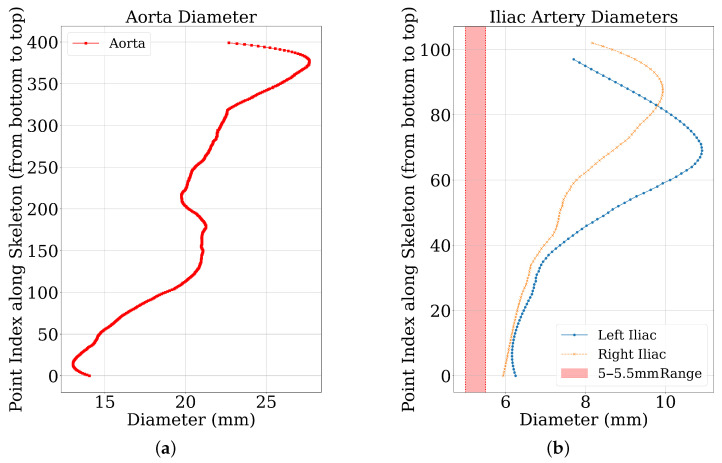
Diameter profiles of a representative sample. (**a**) The diameter measurements along the aorta. (**b**) The diameter measurements for the left and right iliac arteries, with the 5–5.5 mm reference threshold range indicated.

Diameter profiles were obtained from ground truth and predicted segmentation masks using the Section 3.1 pipeline. Figure 4 shows representative profiles. Aortic profiles (Figure 4a) showed characteristic tapering (20–30 mm proximally to 15–25 mm distally). Iliac artery profiles (Figure 4b) were within physiological range (6–12 mm), often including a 5 mm clinical threshold for TAVI access [3]. The y-axis represents the proximal-to-distal point index. These outputs confirm the pipeline’s ability to generate detailed diameter profiles.

### 4.2. Active Learning Performance Evaluation

**Figure 5:**
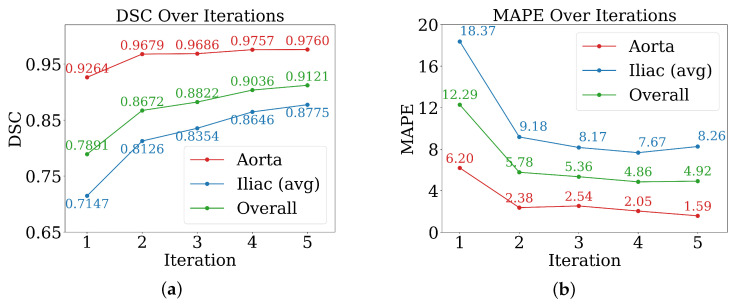
Performance of active learning across iterations for evaluation metrics. (**a**) Dice score; (**b**) mean absolute percentage error (MAPE).

The efficacy of the AL strategy is evaluated until it plateaus for both metrics, DSC and MAPE (Figure 5). Performance plateaued at the fifth iteration, by which point 30 cases had been manually labelled and 60 pseudo-labelled. The remaining 55 cases were kept unseen by the AL model for further evaluation. The DSC improved consistently in all AL iterations (Figure 5a). The iliac arteries, more challenging, showed substantial gains. The overall mean DSC increased from ≈0.789 to 0.912. Most improvements occurred in the initial two to three iterations. Regarding MAPE, it decreased with iterative training (Figure 5b), indicating improved precision. Iliac arteries MAPE reduced from ≈18.37% to 8.26%.

Notably, the steepest performance gains occurred within the first two to three iterations, with DSC improvements of approximately 0.01–0.07 per iteration during this period, compared to marginal gains of 0.008–0.02 in later iterations.

The differential improvement rates between vessel types are also noteworthy: while aortic segmentation achieved high baseline performance (DSC 0.9264) with modest further gains, iliac arteries showed more substantial improvements from 0.7147 to 0.8775 DSC, highlighting the particular value of active learning for anatomically challenging structures.

**Figure 6:**
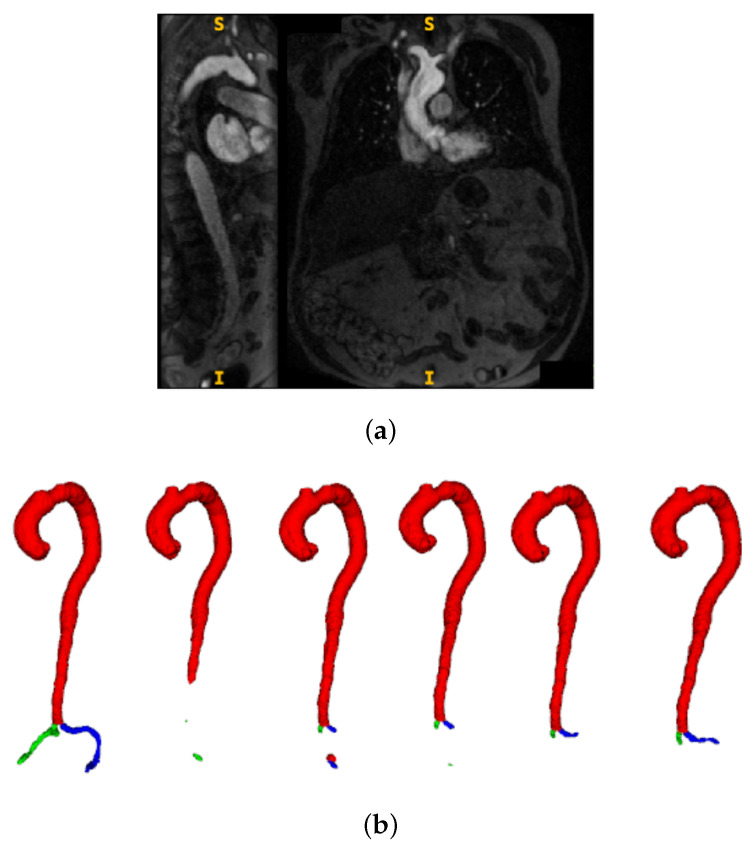
Progressive segmentation improvement across active learning iterations on a challenging test case. (**a**) Input CMR showing coronal and sagittal views with unequal contrast distribution and poor iliac artery visibility. Here, S denotes the superior (upper) direction and I denotes the inferior (lower) direction in the image. (**b**) Segmentation results showing ground truth (leftmost) followed by AL iterations 1–5, demonstrating progressive accuracy improvements particularly in iliac regions.

Figure 6 illustrates the continued learning capability on individual challenging cases even after aggregate performance plateau. This test case exhibits unequal contrast distribution with particularly poor iliac artery visibility (panel a), making accurate segmentation difficult. Despite these challenging conditions, progressive segmentation refinement is evident across all five AL iterations shown in panel (b), with notable improvements in iliac artery delineation compared to ground truth (leftmost in panel b). The qualitative progression reveals enhanced boundary definition and reduced segmentation artifacts from iteration 1 to 5, indicating that the model continues developing robustness for difficult anatomical presentations despite quantitative metric stabilization.

### 4.3. Multimodal Ablation

Table 2 shows the test set DSC for the four different data configurations. The best performance was achieved by 3D U-Net, excluding inferred cases, with an average Dice of 0.887.

Unseen inferred cases reduced performance from 0.887 to 0.877. This may be due to label noise, as these cases were not selected based on high-confidence predictions, unlike the pseudo-labels used during active learning.

**Figure 7:**
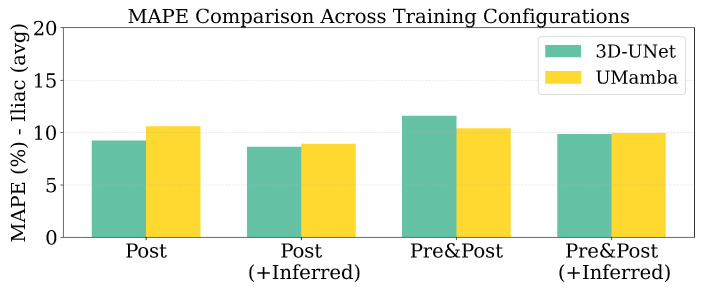
MAPE scores for Iliac diameter across models and data configurations.

Regarding the counterintuitive finding that multimodal input (Pre&Post) did not consistently outperform single-modality (Post-only) configurations, this may indicate alignment challenges between pre- and post-contrast sequences or that the additional information introduces variability that current architectures cannot effectively exploit. Regarding the models used, there was no clear superiority across all configurations. The 3D U-Net showed particular sensitivity to this effect, with multimodal performance dropping to 0.865–0.854 DSC compared to 0.887 for post-only configuration. While 3D U-Net performed better with single-modality input, UMamba slightly outperformed in the multi-modality settings. Figure 7 shows the MAPE across models and data configurations. The lowest error was achieved using only the post-contrast modality with 3D U-Net, consistent with the observed Dice score trends.

## 5. Discussion

This study set out to develop an annotation-efficient active learning framework for vessel segmentation and diameter quantification in support of TAVI access planning. The results demonstrate the feasibility of combining uncertainty-guided case selection with automated diameter measurement, while also highlighting key factors that influence model performance and clinical reliability.

Several important findings emerge from the results:

- Diameter profile validation: The automated pipeline generated physiologically consistent diameter curves (Figure 4), confirming its ability to provide clinically interpretable vessel measurements beyond segmentation accuracy. This is particularly relevant given the 5 mm iliac threshold that often determines transfemoral access eligibility.
- Effectiveness of active learning: Performance improved across iterations (Figure 5), with Dice scores rising from ≈0.79 to 0.91 and iliac MAPE decreasing by more than 50%. The most substantial gains occurred within the first few cycles, showing that expert annotation efforts concentrated on uncertain cases yield rapid improvements, especially for anatomically challenging iliac arteries. The rapid plateau after two to three iterations suggests annotation efficiency can be maximized by focusing expert effort on early cycles, with diminishing returns beyond 20–30 manually labelled cases.
- Impact of modality and pseudo-labels: The multimodal ablation study (Table 2) revealed post-contrast CMR as the most reliable input, delivering the highest Dice and lowest MAPE. By contrast, the inclusion of inferred cases slightly degraded performance, underscoring the sensitivity of segmentation models to label noise when pseudo-label confidence is not tightly controlled. The success of uncertainty-guided pseudo-labelling during active learning contrasted with blind inference performance, emphasizing that pseudo-labelling strategies require carefully designed confidence thresholds and quality control mechanisms.
- Model architecture vs. data quality: Differences between 3D U-Net and UMamba were modest compared to the impact of input modality. This suggests that improving data quality and curation may be more beneficial than pursuing marginal network refinements for this application. Surprisingly, combined pre- and post-contrast data did not consistently improve performance, suggesting current architectures may struggle with effective multimodal feature fusion or that alignment issues introduce artifacts.
- Clinical implications: The combination of high Dice (≈0.97 for aorta, ≈0.85 for iliacs) and low diameter error (≈5% MAPE) supports the potential of this framework for integration into preoperative workflows. Accurate iliac quantification is particularly valuable, as even small diameter misestimations can alter access decisions in borderline cases. While accuracy approaches clinical requirements, results suggest stronger potential for semi-automated workflows where the system provides initial measurements and uncertainty estimates to guide radiologist review. Qualitative analysis (Figure 6) revealed continued improvements for challenging cases even after aggregate metrics plateaued, justifying the full five-iteration protocol.
- Limitations: The study was based on single-center data, which may limit generalizability across populations, scanner vendors, or imaging protocols. Performance on pre-contrast CMR remained substantially weaker, even when combined with post-contrast data, indicating that multimodal integration strategies require further refinement. Finally, as the pipeline is sequential, segmentation inaccuracies propagate to downstream diameter estimation, representing an area for future methodological improvement.

Together, these findings demonstrate that an annotation-efficient active learning pipeline can provide accurate vessel quantification for TAVI planning, while also emphasizing that success depends more on modality choice, label quality, and uncertainty management than on model architecture alone.

## 6. Conclusions

An annotation-efficient active learning (AL) pipeline for CMR-based aortic structure segmentation was presented, addressing data scarcity for TAVI access route planning. This was complemented by an automated workflow for vessel diameter quantification. The AL strategy incorporated uncertainty estimation to guide a hybrid selection of expert-corrected and pseudo-labelled cases.

Experimental results demonstrated the strong performance of this framework, achieving a Dice score of 0.912 for segmentation and a mean absolute percentage error (MAPE) of 4.92% for diameter estimation. The ablation study further revealed that models trained solely on post-contrast CMR consistently outperformed those trained with combined pre- and post-contrast data, suggesting that the additional modality may introduce variability, possibly due to alignment, that negatively impacts model generalization. Furthermore, incorporating inferred cases from the unseen pool did not yield consistent performance gains across configurations. This outcome highlights the critical role of data quality and label reliability in model training. Specifically, the lack of stringent control over the confidence of these pseudo-labels may introduce noise, which can degrade model robustness.

A key contribution of this work is the integration of segmentation and diameter quantification into a complete pipeline for guiding iliac access planning. The final 3D U-Net model trained with post-contrast input demonstrated strong potential for clinical integration, despite the limitation of a single-center study.

Future research should explore more advanced active learning strategies, such as diverse uncertainty metrics or adaptive query methods, to further improve annotation efficiency. In addition, the integration of multimodal data, particularly pre- and post-contrast CMR, represents a promising direction for enhancing segmentation robustness and downstream vessel quantification. Another avenue lies in refining diameter measurement techniques to reduce dependence on upstream segmentation accuracy, for example through optimized centerline extraction or joint optimization frameworks. Finally, multi-center validation studies and domain adaptation strategies will be essential to ensure the generalizability of the proposed framework and to enable its translation into prospective clinical workflows, where its ultimate impact can be assessed in terms of patient outcomes.

## Figures and Tables

**Table 1:** Training configurations.

Trainer	ResEnc M
Model config	3D fullres U-Net
Total epochs	300
Optimizer	SGD
Initial learning rate (lr)	1 × 10^−2^
Momentum	0.99
Weight decay	3 × 10^−5^
Lr decay schedule	PolyLR
Loss function	Dice + Cross-Entropy
Deep supervision	True
Oversample foreground (%)	0.33
Probabilistic oversampling	False
Gradient clipping norm	12

**Table 2:** Dice scores for aorta and iliac segmentation across data configurations. Notes: For each metric, the single best result across all configurations is highlighted in **bold**. Results within 0.005 of the best score are underlined. Blue text indicates pre-contrast dataset, while brown text marks configurations that include inferred predictions.

Configuration	Model	Aorta	Left Iliac	Right Iliac	Average
Post	3D-UNet	0.9664	0.8250	**0.8627**	**0.8871**
	UMamba	0.9678	0.8111	0.8321	0.8732
Post + Inferred	3D-UNet	0.9626	0.8107	0.8507	0.8773
	UMamba	0.9617	**0.8272**	0.8260	0.8743
Pre&Post	3D-UNet	0.9667	0.8167	0.8132	0.8655
	UMamba	**0.9684**	0.8222	0.8404	0.8770
Pre&Post + Inferred	3D-UNet	0.9677	0.7991	0.7954	0.8541
	UMamba	0.9660	0.8152	0.7944	0.8585

## Data Availability

The data that support the findings of this study are available from the corresponding author upon reasonable request. Access is restricted due to privacy and ethical considerations.
